# Autophagy-related tumor subtypes associated with significant gene expression profiles and immune cell infiltration signatures to reveal the prognosis of non-small cell lung cancer

**DOI:** 10.7150/jca.83097

**Published:** 2023-05-15

**Authors:** Rong-Ai Wang, Meng-Yu Zhang, Ying-Xiao Jiang, Xiao-Dong Wang, Jia-Jia Qu, Yue-Liang Yue, Yi-Qing Qu

**Affiliations:** 1 The Second Affiliated Hospital of Zhejiang Chinese Medical University, Hangzhou, Zhejiang, China.; 2 Qilu Hospital of Shandong University, Jinan, Shandong, China.; 3 Department of Pulmonary and Critical Care Medicine, Laboratory of Basic Medical Sciences, Qilu Hospital of Shandong University, Jinan, Shandong, China.

**Keywords:** non-small cell lung cancer, autophagy, prognostic subtype, mutation, immune cell infiltration

## Abstract

Autophagy plays an important role in non-small cell lung cancer (NSCLC). We aimed to establish novel autophagy-related tumor subtypes to distinguish the prognosis of NSCLC. In this study, gene expression profiles, mutation data and clinical information obtained from the Cancer Genome Atlas. Kaplan Meier-plotter could evaluate prognostic value of autophagy-related genes. Consensus clustering revealed autophagy-related tumor subtypes. Gene expression profiles, mutation data and immune infiltration signatures were identified, oncogenic pathways and gene-drug interactions were performed according to the clusters. Finally, a total of 23 prognostic genes were screened and consensus clustering analysis divided the NSCLC into 2 clusters. The mutation signature showed that 6 genes are special. Immune infiltration signatures showed that higher fraction of immune cells was associated with cluster 1. The oncogenic pathways and gene-drug interactions also showed different patterns. In conclusion, autophagy-related tumor subtypes have different prognosis. Understanding the subtypes of NSCLC are helpful to accurately identify the NSCLC and personalized treatment.

## Introduction

Lung cancer remains the leading cause of cancer-related mortality worldwide, in which non-small cell lung cancer (NSCLC) account for nearly 85% of all the lung cancer 1,2. Lung adenocarcinoma (LUAD) and lung squamous cell carcinoma (LUSC) are the main type of NSCLC 3,4. There are improvements of therapy methods, such as chemotherapeutic drugs and immune therapy, while the 5-year survival rate of NSCLC patients is only 18% 5. Furthermore, surgical resection is the most beneficial treatment for NSCLC, but most newly diagnosed patients are at the onset of advanced or metastatic stages and usually lost the chance for operation. For the clinical tumor-node-metastasis (TNM) stage IIIB NSCLCs, the 5-year survival rate is only 7%, and the 5-year survival rate of TNM stage IV NSCLC patients as low as 2% 6. Hence, it is essential to manage the patients according to the biomarkers for early detection of NSCLC in order to improve the prognosis and reduce the mortality rates.

Autophagy is a key biological process, it could maintain the cellular homeostasis by engulfing cytoplasmic proteins, complexes or organelles within the autophagosome 7,8. Autophagosome is a cytoplasmic double-membrane and it can be transported and fused with lysosome to generate the autolysosome 9. Autophagy was reported to associated with tumorigenesis 10,11. Over the past few years, a lot of studies have elucidated that autophagy take part in the development and progression of NSCLC 12,13. In brief, autophagy has dual functions in the tumorigenesis, including positive and negative effects. Positive parts behaved as proper degree of autophagy could clear damaged proteins and organelles in the early stages of the tumor so as to inhibiting tumor development 14. Negative effects are involved in the advanced stages of tumorigenesis, autophagy could promote rapid growth of tumor cells by degrading and recycling the damaged or aged organelles components 15.

Up to date, there are increasing evidence indicated the role of autophagy-related genes in the development of cancer. Autophagy-related gene expression signature could serve as an independent prognostic indicator for serous ovarian cancer 16. Eight autophagy-related genes (BCL2, BIRC5, EIF4EBP1, ERO1L, FOS, GAPDH, ITPR1 and VEGFA) were explored and the author found that these genes are significantly associated with overall survival in breast cancer 17. The Beclin1 and LC3 genes were correlated with the tumor stage, metastasis conditions and mortality in pancreatic cancer 18. There is also another one study proposed an autophagy-related gene prognostic signature and divided all the patients into high-risk and low-risk groups and the author concluded that autophagy-related gene prognostic signature is a promising biomarker for monitoring the outcomes of LUAD and LUSC 19. These findings confirm the role of autophagy in cancers and suggest that autophagy-related genes maybe served as prognostic biomarkers.

Although there are many studies focus on the relationship between autophagy-related gene expression signature and the prognosis of cancer, very few studies have studied the reason why the autophagy-related gene expression signature could influence the prognosis of NSCLC. The purpose of this study was to establish novel autophagy-related tumor subtypes to predict the prognosis of NSCLC. Meanwhile, we also want to explain possible reasons why the novel autophagy-related tumor subtypes could influence the prognosis of NSCLC.

## Materials and methods

### Data collection

The gene expression profiles, mutation data and clinical information of NSCLC patients were downloaded from the Cancer Genome Atlas (TCGA) database (https://tcga-data.nci.nih.gov/tcga/). In detail, TCGA contains a total of 1102 patients (including 103 adjacent normal lung tissues and 999 NSCLC tissues). The selected criteria were the followings: gene expression profiles, mutation expression profiles, studies compared adjacent non-tumorous lung tissues and NSCLC tissues in human. The excluded criteria were the followings: those studies that compared genes between lung cancer and benign disease in human, expression profiles using cell lines or serum, saliva, peripheral blood; patient had no survival time or survival status, patient had clinical information but no gene expression data. After screening according to the criteria, there are 990 NSCLC patients left, including 486 LUAD patients and 504 LUSC patients (Table 1). The Human Autophagy Database (HADb; http://www.autophagy.lu) is the first human autophagy-dedicated database, it is a public repository containing information about the human genes described so far as involved in autophagy. A total of 232 genes from the HADb were identified as autophagy-related genes.

### Identification of differentially expressed autophagy-related genes

Gene expression data from TCGA was analyzed by the R package limma package. The cut-off criterion was set as the p < 0.05 and absolute fold change > 2. In addition, the R package ggplot2 package was used to perform the volcano plots of all the autophagy-related genes between adjacent normal lung tissues and NSCLC tissues. Heat maps for the differentially expressed autophagy-related genes was generated using the R package pheatmap package. Then Kaplan Meier-plotter (https://kmplot.com/analysis/) was used to evaluate the prognostic value of differentially expressed autophagy-related genes in NSCLC.

### Functional enrichment analysis and protein-protein interaction (PPI) network construction

Kyoto Encyclopedia of Genes and Genomes (KEGG) is a knowledge base for systematic analysis of gene functions. Gene ontology (GO) enrichment analysis predicts the function of the target genes in three aspects, including biological processes, cellular components and molecular function. There are several ways to performed the functional enrichment analysis and clusterProfiler package was used in our study 20. P<0.05 was the threshold for the identification of significant GO terms and KEGG pathways. The GOplot package was employed to visualize the enrichment terms. PPI network was constructed by the STRING database (https://string-db.org) 21 and cytoscape software 22.

### Evaluation of tumor-infiltrating immune cells

CIBERSORT is an algorithm that uses gene expression data to quantify specific cell types in mixed cell populations 23. The CIBERSORT method is used to estimate the score of immune cells in LUAD and LUSC samples. The normalized gene expression data were prepared using standard annotation files and data were uploaded to the CIBERSORT, then the R package Genefilter package was utilized to screen each LUAD and LUSC sample. With the threshold of p<0.05, the result of the inferred score of immune cell populations were considered accurate.

### Statistical analysis

All statistical analysis was performed using the R software, and p<0.05 was regarded as statistically significant. The unpaired t test was used to assess the expression level of the autophagy-related genes between cluster 1 and cluster 2. The Kaplan-Meier survival curve analysis and the log-rank test were used to analyze overall survival. The difference of infiltrating immune cells between cluster 1 and cluster 2 was assessed by unpaired t test.

## Results

### Identification of differentially expressed autophagy-related genes and their prognostic value in NSCLC

**Figure 1:**
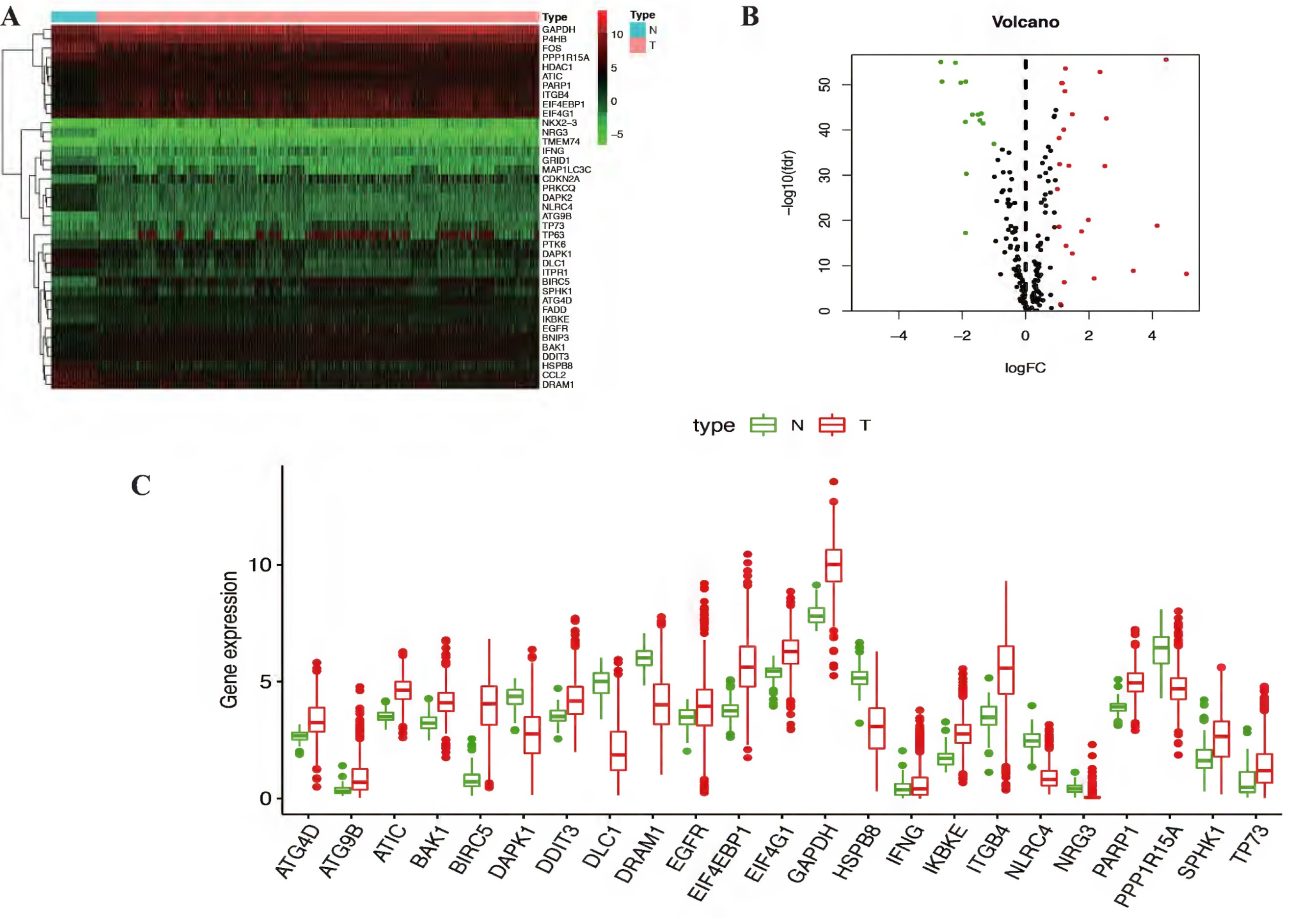
** Identification of differentially expressed autophagy-related genes in NSCLC.** (A) Two-dimensional hierarchical clustering of the significant 39 differentially expressed autophagy-related genes in all samples. Genes are in rows; samples are in columns. Both up-regulated and down-regulated autophagy-related genes can be seen in tumors compared with normal tissues. (B) Volcano plots of the 232 autophagy-related genes analysis. There were 39 genes identified to be differentially expressed, including 14 down-regulated genes and 25 up-regulated genes. X-axis: log 2-fold change; Y-axis: -log10 fdr for each probe. (C) The boxplot of the differentially expressed autophagy-related genes. The red color indicates tumor tissues and the green color indicated the non-tumor. NSCLC, non-small cell lung cancer.

In this study, gene expression profiles from TCGA database in NSCLC were selected and the expression levels of differentially expressed autophagy-related genes were extracted. Genes with p<0.05 and absolute fold change>2 were considered as differentially expressed autophagy-related genes. A total of 232 autophagy-related genes were pooled from HADb. After screening process, there are 39 autophagy-related genes were differentially expressed in NSCLC, including 14 down-regulated genes and 25 up-regulated genes (Table 2) (Figure 1A-B). Then Kaplan Meier-plotter online database was used to evaluate the prognostic value of 39 differentially expressed autophagy-related genes in NSCLC, and the results showed that only 23 differentially expressed autophagy-related genes (Figure 1C) were related to the overall survival for NSCLC, including 7 down-regulated genes and 16 up-regulated genes (Figure S1).

### Functional enrichment analysis of differentially expressed autophagy-related genes

**Figure 2:**
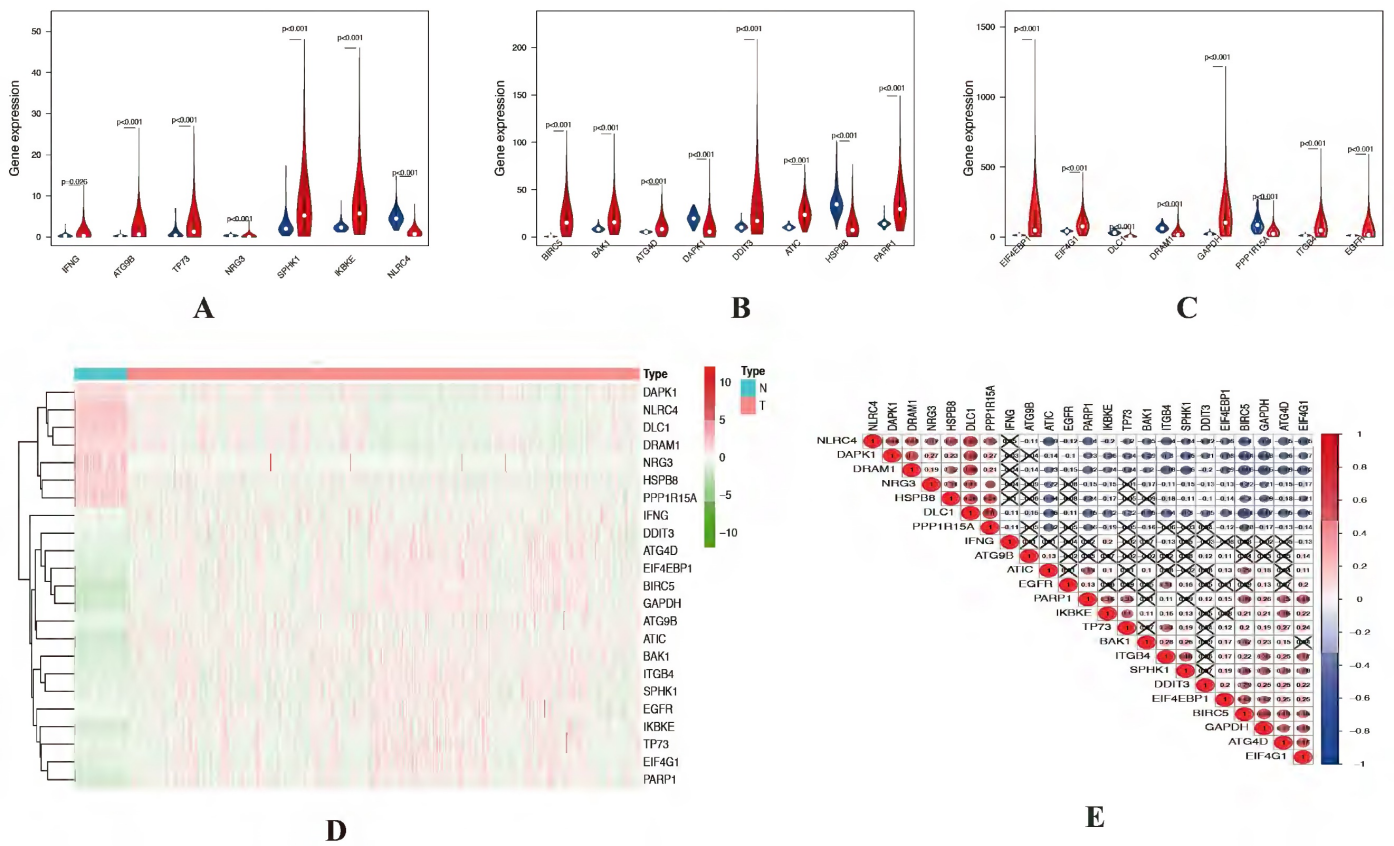
** Expression levels of differentially expressed autophagy-related genes. (**A-C) Violin plots of 23 prognostic autophagy-related gene expression levels. X-axis: gene name; Y-axis: gene expression. (D) Two-dimensional hierarchical clustering of the significant 23 prognostic autophagy-related genes in all samples. Genes are in rows; samples are in columns. Both up-regulated and down-regulated autophagy-related genes can be seen in tumors compared with normal tissues. (E) Correlation of 23 prognostic differentially expressed autophagy-related genes.

**Figure 3:**
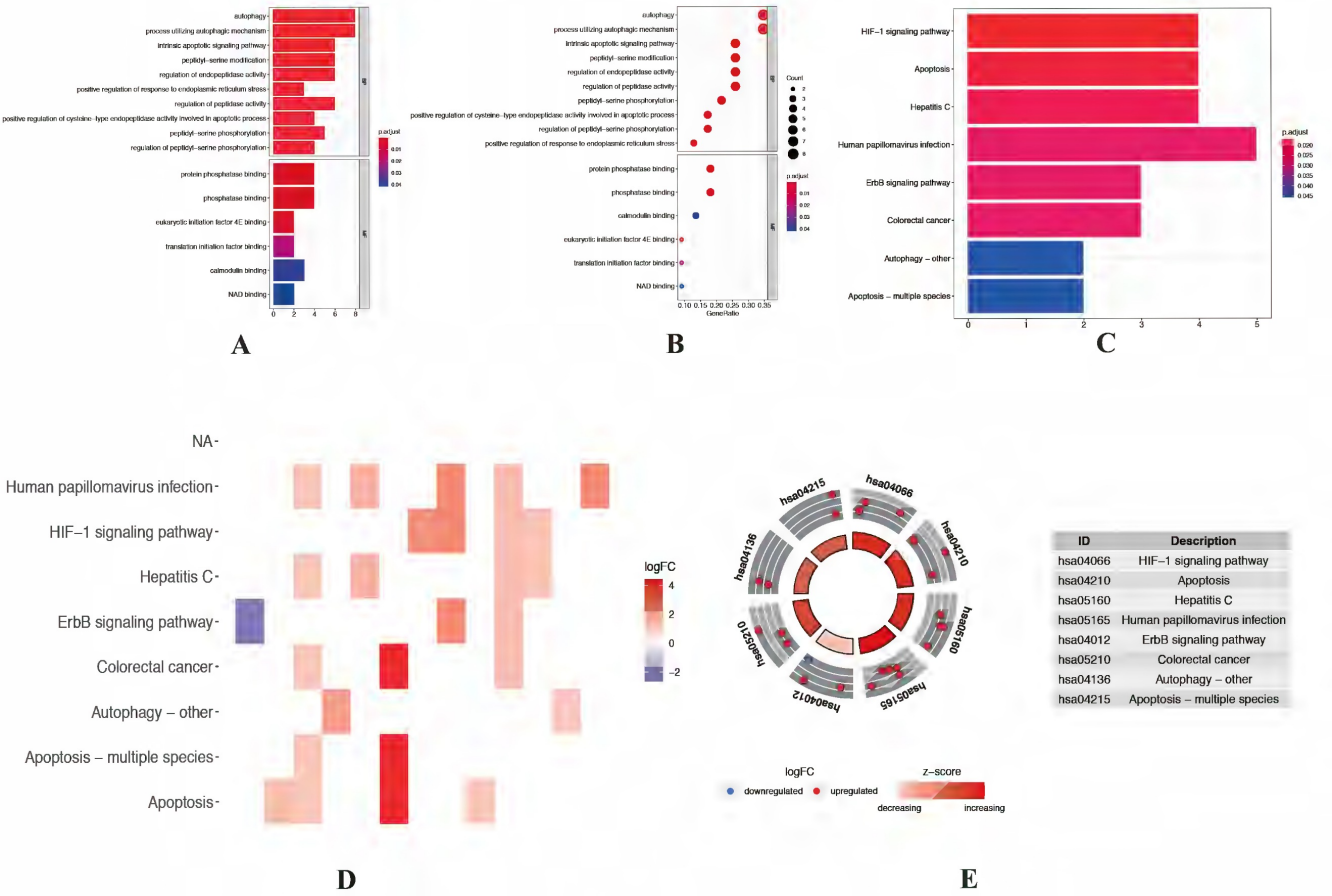
** Functional enrichment analysis of differentially expressed autophagy-related genes.** (A-B) The bar plot and bubble plot of significant GO terms. (C-D) The bar plot and heat map of enriched KEGG pathways. (E) KEGG circle of functional enrichment analysis of differentially expressed autophagy-related genes. The red circles display up-regulation, and the blue ones display down-regulation. The higher the Z-score value indicated, the higher expression of the enriched pathway. GO, Gene Ontology; KEGG, Kyoto Encyclopedia of Genes and Genomes.

The expression levels of 23 prognostic differentially expressed autophagy-related genes were visualized by violin plots (Figure 2A-C) and heatmap (Figure 2D). Besides, correlation of 23 prognostic differentially expressed autophagy-related genes were also explored (Figure 2E). To determine biological functions of the prognostic 23 differentially expressed autophagy-related genes, gene ontology (GO) enrichment analysis was performed to predict the function of the 23 differentially expressed autophagy-related genes in biological processes, cellular components and molecular function. The results showed that the identified differentially expressed autophagy-related genes were mainly involved in biological processes were autophagy, process utilizing autophagic mechanism, intrinsic apoptotic signaling pathway, peptidyl-serine modification and regulation of endopeptidase activity. The most significantly enriched molecular function concentrated on protein phosphatase binding, phosphatase binding, eukaryotic initiation factor 4E binding, translation initiation factor binding and calmodulin binding. It seems differentially expressed autophagy-related genes have no significant relationship to CC according to the p value (Figure 3A-B). Further Kyoto Encyclopedia of Genes and Genomes (KEGG) analysis was also performed to investigate the significance of differentially expressed autophagy-related genes in the development of NSCLC. The result showed that 23 differentially expressed autophagy-related genes were enriched in 8 KEGG pathways (Figure 3C-E). According to the results of functional enrichment of differentially expressed autophagy-related genes, we found that differentially expressed autophagy-related genes was not only connected to autophagy but also involved in other biological processes. So, in this study, we hope to identify autophagy subtypes different biological characters based on 23 differentially expressed autophagy-related genes associated with of NSCLC.

### Consensus clustering and principal components analysis of differentially expressed autophagy-related genes identified two clusters of NSCLC

**Figure 4:**
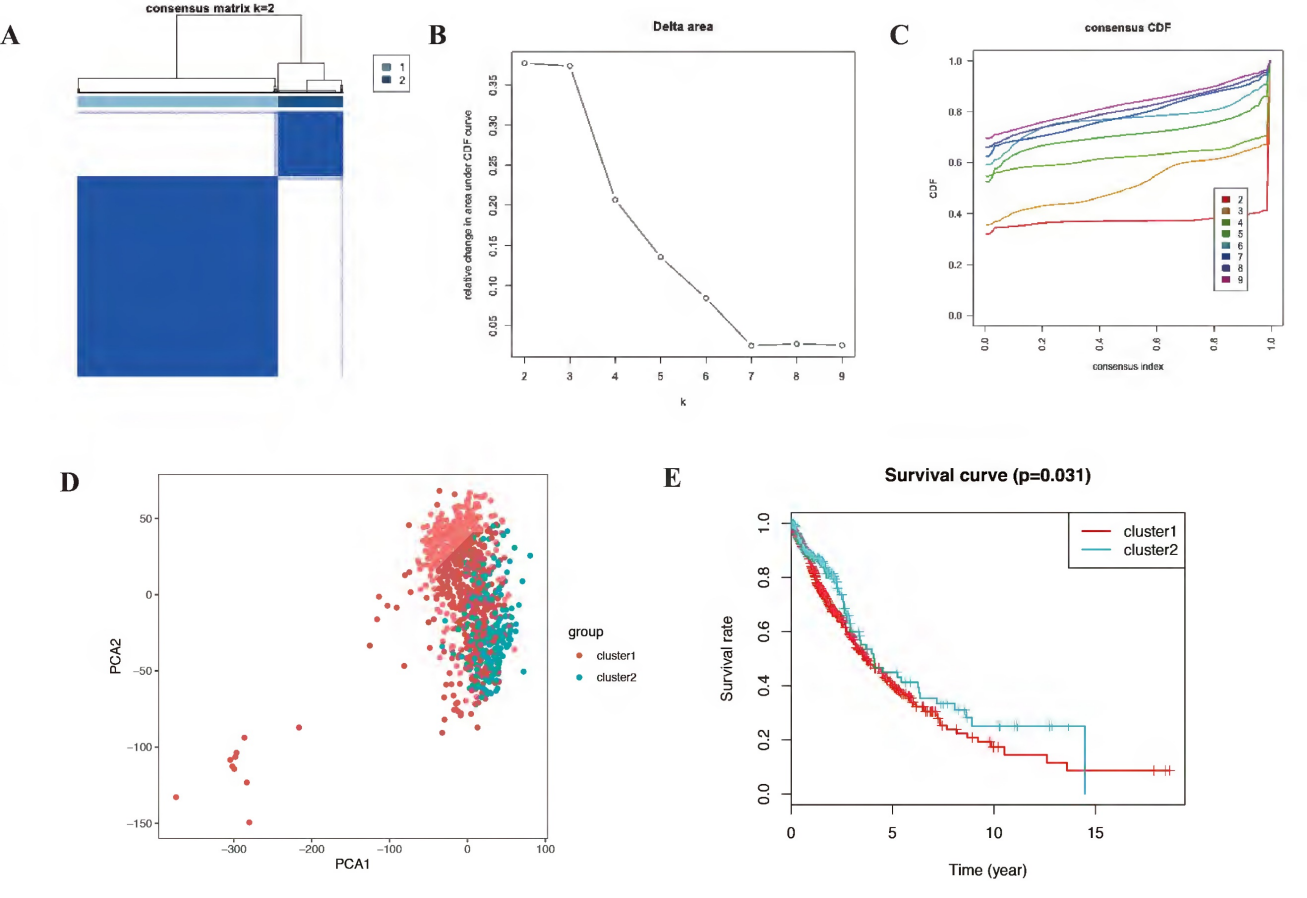
** The consensus clustering analysis and the principle components analysis.** (A-C) The consensus clustering analysis of the prognostic autophagy-related genes, inferring the optimal number of clusters, the lowest proportion of ambiguous clustering and the best CDF value by taking the K value of 2. (D) The principle components analysis of the prognostic autophagy-related genes in NSCLC patients. E, Kaplan-Meier analysis of cluster 1 and cluster 2. CDF, cumulative distribution function; NSCLC, non-small cell lung cancer.

Autophagy may have different expression patterns among NSCLC patients, which partially affects the prognosis and gene expression signature. In this study, 23 differentially expressed autophagy-related genes were used to identify autophagy subtypes associated with overall survival of NSCLC. Consensus clustering was used to explore the similarity of 23 differentially expressed autophagy-related gene expression patterns. By selecting k value of 2, we obtained the optimal cumulative distribution function (CDF) value and classified the NSCLC patients into 2 clusters (Figure 4A-C). Principal components analysis (PCA) revealed two significantly different distribution patterns of NSCLC patients. The samples of cluster 1 and cluster 2 were distributed on the left side and right side, respectively (Figure 4D). Consensus clustering and principal components analysis suggested that autophagy may plays a role in the occurrence and development of NSCLC. Besides, to explore whether these 2 clusters will affect the clinical outcomes, we constructed a prognostic classifier using Kaplan-Meier analysis. The results showed that the prognosis of cluster 2 expression pattern is better than cluster 1 (p = 0.031) (Figure 4E). In detail, the five-year overall survival rate in cluster 2 (43.2%) is better than cluster 1 (40.4%), cluster 2 also have better ten-year overall survival rate (29.1%) than cluster 1 (20.4%). There is a difference in fifteen-year overall survival for cluster 1 have 10.4% fifteen-year overall survival, while there is no clue about cluster 2. Besides, we also noticed that the survival curves of these two clusters crossed before year fifteen, which mean there are other factors affect prognosis and it should be further discussed in the future.

### Identification of differentially expressed genes (DEGs) and functional enrichment analysis between cluster 1 and cluster 2

**Figure 5:**
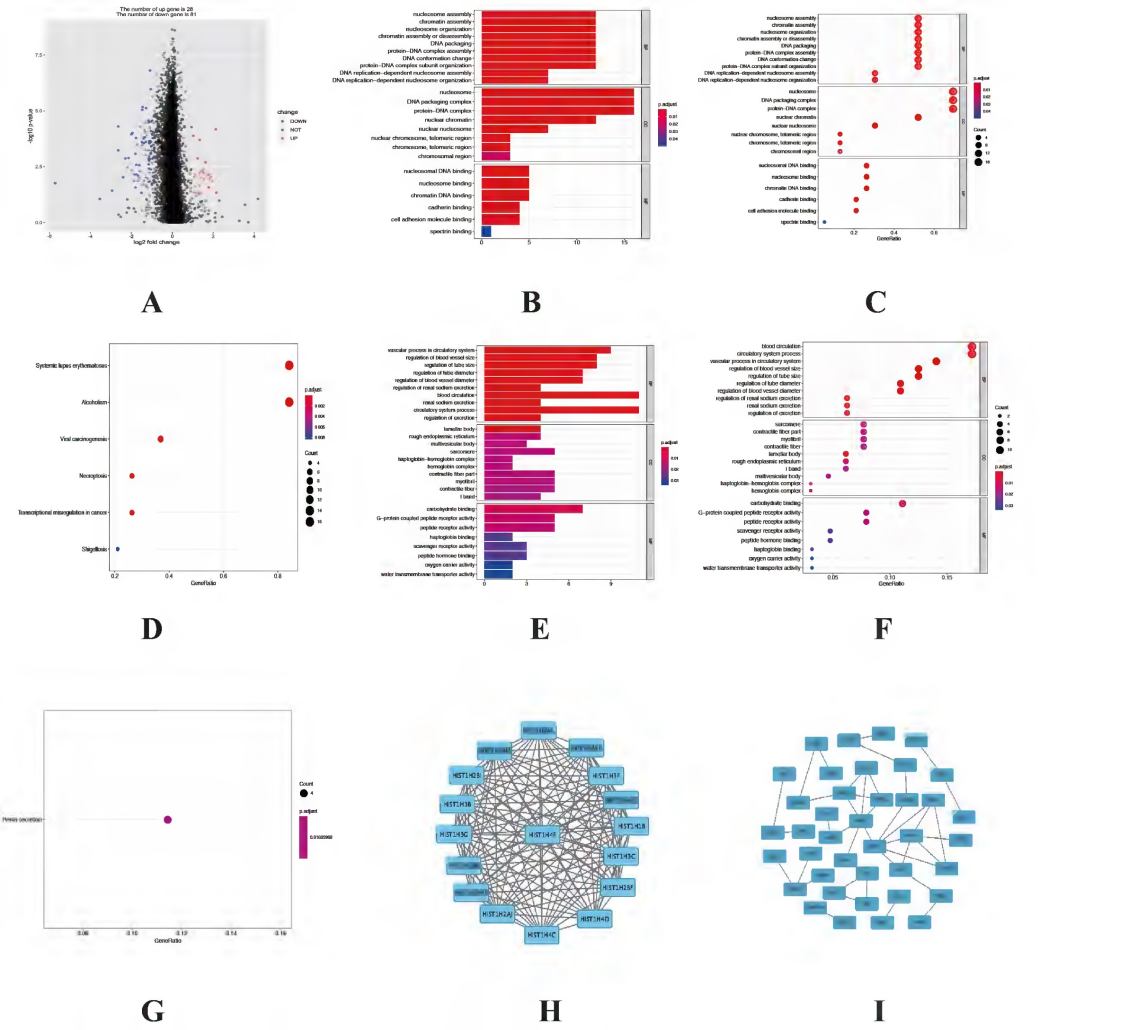
** Functional enrichment analysis of DEGs between cluster 1 and cluster 2.** (A) Volcano plot of the 109 DEGs, including 28 up-regulated genes in cluster 1 and 81 up-regulated genes in cluster 2. (B-C) The bar plot and bubble plot of significant GO terms from 28 up-regulated genes in cluster1. (D) The bubble plot of enriched KEGG pathways from 28 up-regulated genes in cluster1. (E-F) The bar plot and bubble plot of significant GO terms from 81 up-regulated genes in cluster2. (G) The bubble plot of enriched KEGG pathways from 81 up-regulated genes in cluster2. (H-I) PPI networks of the DEGs from cluster 1 and cluster 2, respectively. DEGs, differentially expressed genes; GO, Gene Ontology; KEGG, Kyoto Encyclopedia of Genes and Genomes; PPI, Protein-protein interaction.

Since different clusters have shown variations in the autophagy-related genes and patient prognosis, we explored the DEGs in the cluster 1 and cluster 2. A total of 109 DEGs (28 up-regulated genes in cluster 1 and 81 up-regulated genes in cluster 2) were screened and visualized (Figure 5A). To explore biological functions of the prognostic value of DEGs from these 2 clusters, GO and KEGG enrichment analysis were performed. The results showed the identified DEGs in cluster 1 mainly involved in biological processes were nucleosome assembly, chromatin assembly and nucleosome organization. The most significantly enriched cellular components were nucleosome, DNA packaging complex and protein-DNA complex. As for molecular function, which were statistically concentrated on nucleosomal DNA binding, nucleosome binding and chromatin DNA binding (Figure 5B-C). KEGG analysis result showed that DEGs in cluster 1 were enriched in six pathways, including systemic lupus erythematosus, alcoholism, viral carcinogenesis, necroptosis, transcriptional misregulation in cancer and shigellosis (Figure 5D). While the functional analysis results in cluster 2 showed the DEGs mainly involved in vascular process in circulatory system, regulation of blood vessel size and regulation of tube size in biological processes. While in cellular components the DEGs mainly enriched in lamellar body, rough endoplasmic reticulum and multivesicular body. As for molecular function, the DEGs mainly involved in carbohydrate binding, G-protein coupled peptide receptor activity as well as peptide receptor activity (Figure 5E-F). The KEGG analysis result showed that the DEGs were only enriched in renin secretion (Figure 5G). Besides, the STRING database and Cytoscape software were used to construct the PPI networks for DEGs of each cluster (Figure 5H-I).

### Identification of the significant mutation profile signature between cluster 1 and cluster 2

**Figure 6:**
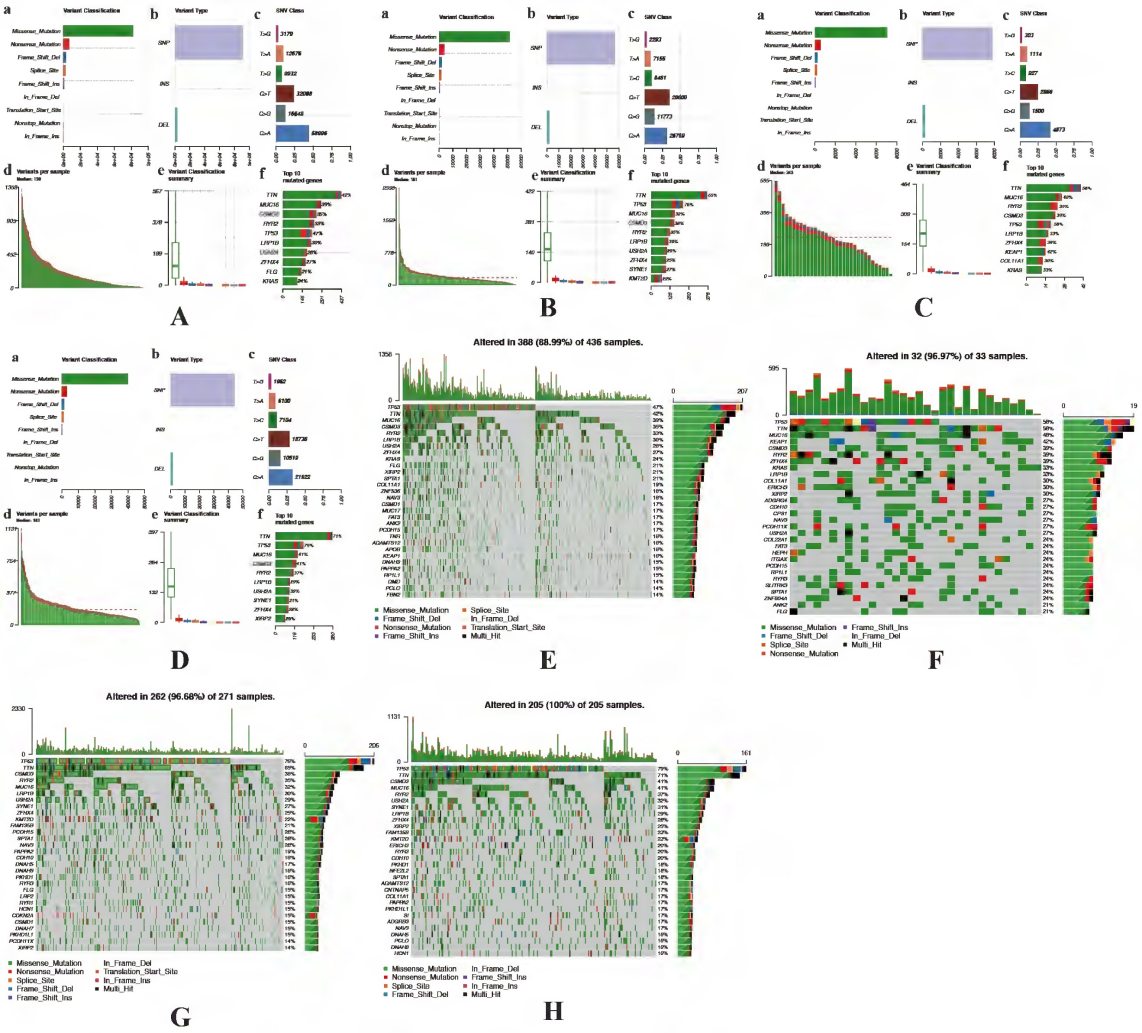
** Identification of mutation profile signature in cluster 1 and cluster 2 of LUAD and LUSC.** Summary of mutation data in cluster 1 (A) and cluster 2 (B) of LUAD. Summary of mutation data in cluster 1 (C) and cluster 2 (D) of LUSC. E-H, Driver gene mutations in different autophagy-related subtypes of NSCLC. The top panel shows the mutation rates (number of mutations) per patient in cluster 1 (E, G) and cluster 2 (F, H) of LUAD and LUSC, respectively. LUAD, lung adenocarcinoma; LUSC, lung squamous cell carcinoma; NSCLC, non-small cell lung cancer.

The accumulation of somatic DNA mutation plays an important role in the formation of tumor. In this study, we explored the difference of mutation profile signatures for LUAD and LUSC based on the different prognosis between cluster 1 and cluster 2. In these 2 clusters, the proportion of missense mutations is the major mutation, the most variant type is SNP both in LUAD and LUSC. In the cluster 1, C > A (58996) is the highest mutation mode of SNP in LUAD (Figure 6A), while C > A (29600) is the main mutation mode of SNP in LUSC (Figure 6B). The average number of mutations in each sample is 136 in LUAD and 181 in LUSC. As for cluster 2, C > A (4873 and 58996) is the highest mutation mode of SNP in LUAD and LUSC, the average number of mutations in each sample is 243 in LUAD and 183 in LUSC (Figure 6C-D).

**Figure 7:**
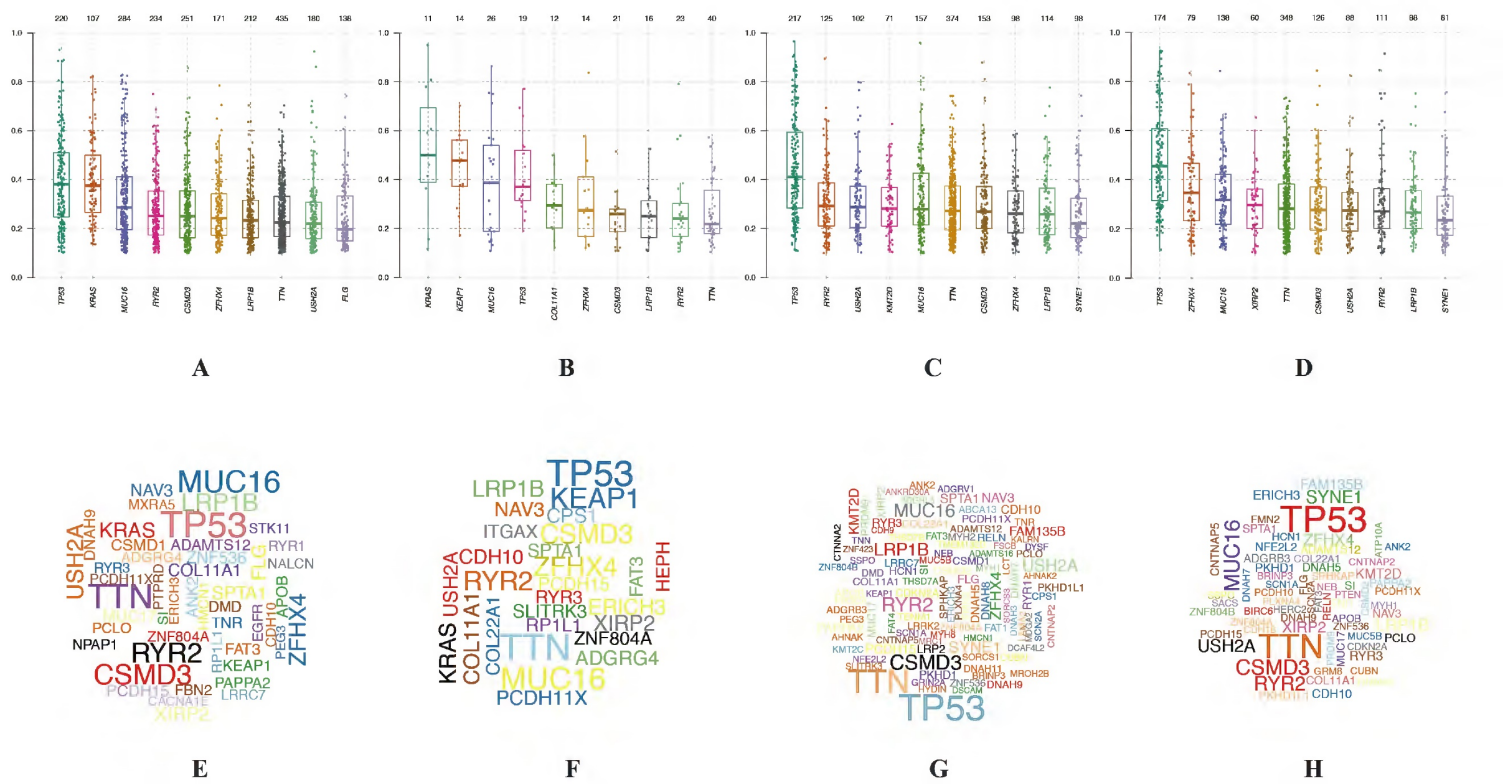
** Visualization of mutation profile signature in cluster 1 and cluster 2 of LUAD and LUSC.** Variant allele frequency (VAF) in cluster 1 (A, C) and cluster 2 (B, D) of LUAD and LUSC, respectively. Gene cloud maps of mutant genes in cluster 1 (E, G) and cluster 2 (F, H) of LUAD and LUSC, respectively. Gene names size is proportional to the number of samples each gene mutation. LUAD, lung adenocarcinoma; LUSC, lung squamous cell carcinoma.

**Figure 8:**
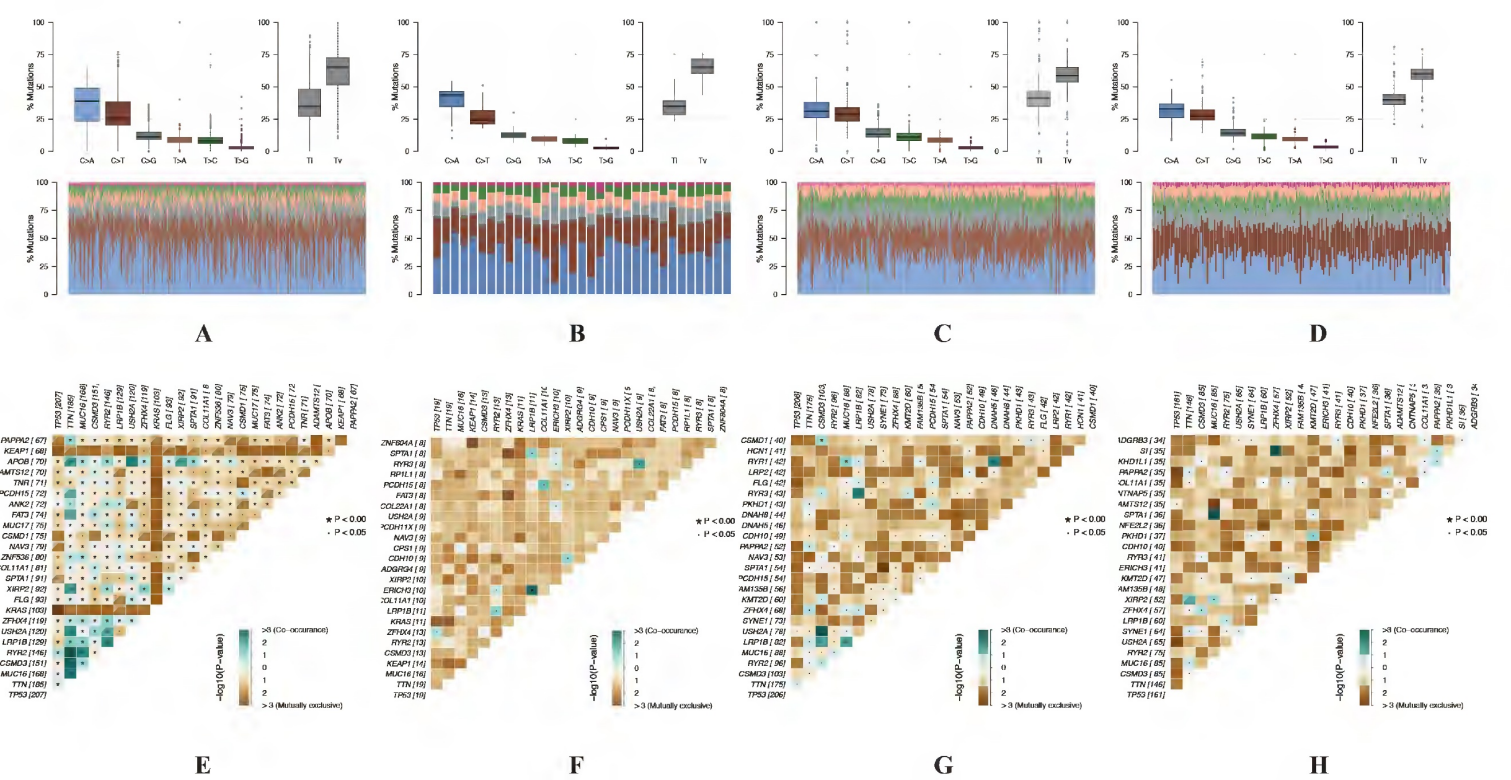
** Visualization of mutation profile signature in cluster 1 and cluster 2 of LUAD and LUSC.** Mutation mode of SNP in cluster 1 (A, C) and cluster 2 (B, D) of LUAD and LUSC, respectively. Somatic interactions of significant mutated genes in cluster 1 (E, G) and cluster 2 (F, H) of LUAD and LUSC, respectively. LUAD, lung adenocarcinoma; LUSC, lung squamous cell carcinoma.

In LUAD, 8 genes (TTN, MUC16, CSMD3, RYR2, TP53, LRP1B, ZFHX4 and KRAS) were mutated in both cluster 1 and cluster 2 among top 10 mutated genes, which means that the mutation rate of these 8 genes have no significant difference between cluster 1 and cluster 2. While the mutated FLG (21%) and USH2A (28%) are special for cluster 1 (Figure 6E), meanwhile the mutated KEAP1 (42%) and COL11A1 (30%) are special for cluster 2 (Figure 6F). In LUSC, 9 of top 10 mutated genes were consistent in these 2 clusters, while KMT2D (22%) are special for cluster 1 and XIRP2 (25%) are special for cluster 2 (Figure 6G-H). Besides, the most significant mutated genes (Figure 7), mutation mode of SNP (Figure 8A-D), and the somatic interactions among these significant mutated genes were also visualized (Figure 8E-H).

### Identification of immune cell infiltration signatures of each cluster based on tumor mutation burden (TMB)

**Figure 9:**
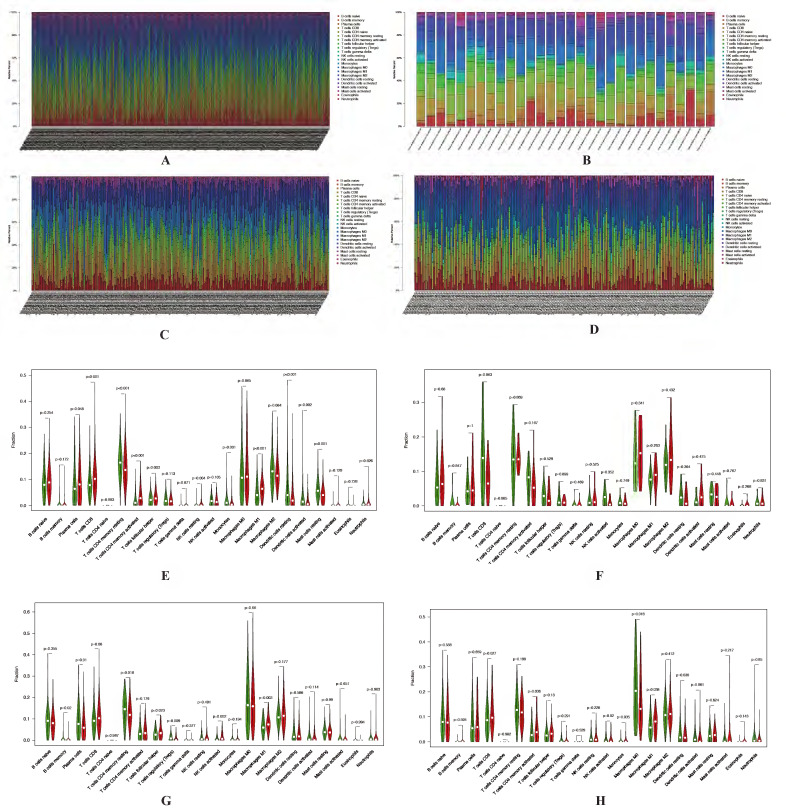
** Identification of immune cell infiltration signatures of each cluster.** The percent of 22 types of fractions of tumor infiltrating immune cell in cluster 1 (A) and cluster 2 (B) of LUAD. The percent of 22 types of fractions of tumor-infiltrating immune cell cluster 1 (C) and cluster 2 (D) of LUSC. Violin plots of immune cell infiltration signatures in cluster 1 (E, G) and cluster 2 (F, H) of LUAD and LUSC, respectively. LUAD, lung adenocarcinoma; LUSC, lung squamous cell carcinoma.

A lot of studies have showed that autophagy was involved in tumor microenvironment, and the autophagy-related genes could affect the immune responses. Based on the different prognosis between these 2 clusters and different TMB level for the samples in each cluster, we explored the difference of immune cell infiltration signatures for each cluster in both LUAD and LUSC according to the TMB levels. The fractions of infiltrating immune cells in tumor tissue were calculated by CIBERSORT algorithm. The cut-off of p value is 0.05. In LUAD, there are 436 patients which including 205 low TMB samples and 198 high TMB samples in cluster 1. In cluster 2, there are 17 low TMB and 13 high TMB samples. In LUSC, there are 128 and 97 low TMB samples, 124 and 94 high TMB samples in cluster 1 and cluster 2, respectively. Immune cell infiltration signature of cluster 1 and cluster 2 both in LUAD and LUSC patients were displayed as boxplots (Figure 9A-D).

The results shown that cluster 1 of LUAD have higher fraction of T cells CD8 (p < 0.001), T cells CD4 memory resting (p < 0.001), T cells CD4 memory activated (p < 0.001), Macrophages M1 (p < 0.001), Dendritic cells resting (p < 0.001), Mast cells resting (p < 0.001), Monocytes (p = 0.001), Dendritic cells activated (p = 0.002), T cells follicular helper (p = 0.003), NK cells resting (p = 0.004) and Plasma cells (p = 0.048) (Figure 9E-F). As for LUSC, both in cluster 1 and cluster 2 have higher fraction of Macrophages M1 (p = 0.003 and p = 0.038). Cluster 1 have a higher fraction of B cells memory (p = 0.020), Plasma cells (p = 0.010), T cells CD4 memory resting (p = 0.018), T cells follicular helper (p = 0.023), T cells regulatory (Tregs) (p = 0.009) and NK cells activated (p = 0.002). Higher fraction of Monocytes (p = 0.035) and Macrophages M0 (p = 0.018) seems associated with cluster 2 (Figure 9G-H).

### Significant oncogenic pathways and drug-gene interactions between cluster 1 and cluster 2

**Figure 10:**
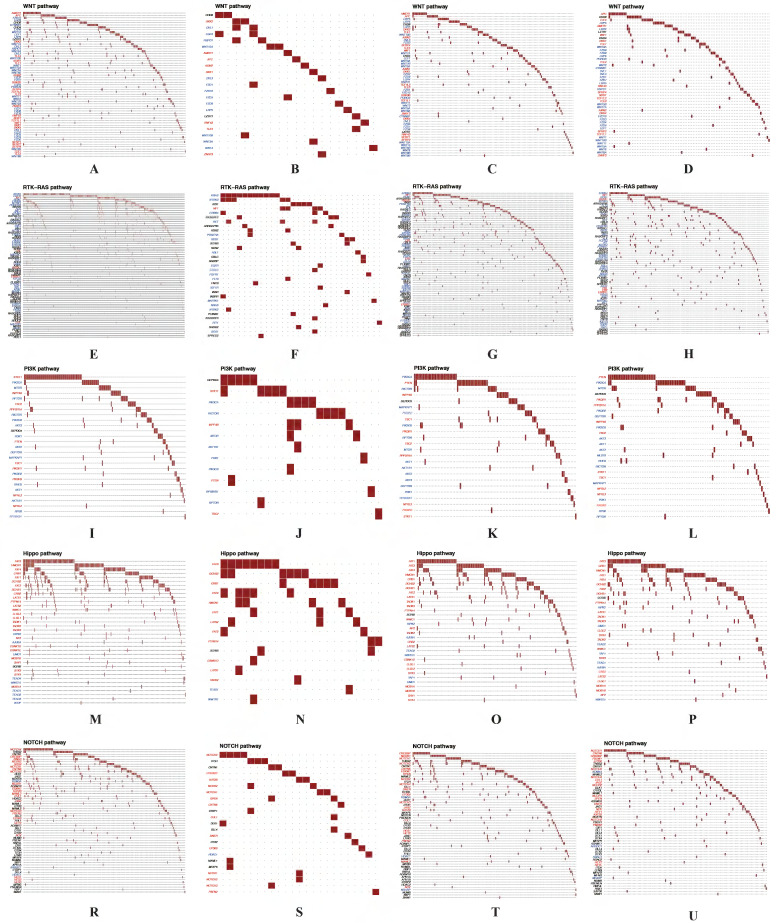
** Significant oncogenic pathways and drug-gene interactions in each cluster.** The mutated oncogenic pathways are mainly involved in WNT (A-D), RTK-RAS (E-H), PI3K (I-L), Hippo (M-P) and NOTCH (R-U) signaling pathways both in cluster 1 and cluster 2 of LUAD and LUSC, respectively. LUAD, lung adenocarcinoma; LUSC, lung squamous cell carcinoma.

For the DEGs from different clusters, significant mutation profile signature from LUAD and LUSC were screened. Besides, we also explored the oncogenic pathways between cluster 1 and cluster 2. After we visualized all the oncogenic pathways, we found that the mutated oncogenic pathways are mainly involved in WNT, RTK-RAS, PI3K, NOTCH and Hippo signaling pathways both in LUAD and LUSC according to the cluster classification (Figure 10). The most significant difference of the same mutated signaling pathway between cluster 1 and cluster 2 is the numbers of mutated genes and mutated sample numbers. It seemed that cluster 1 have more mutated samples and genes compared to cluster 2 both in LUAD and LUSC.

**Figure 11:**
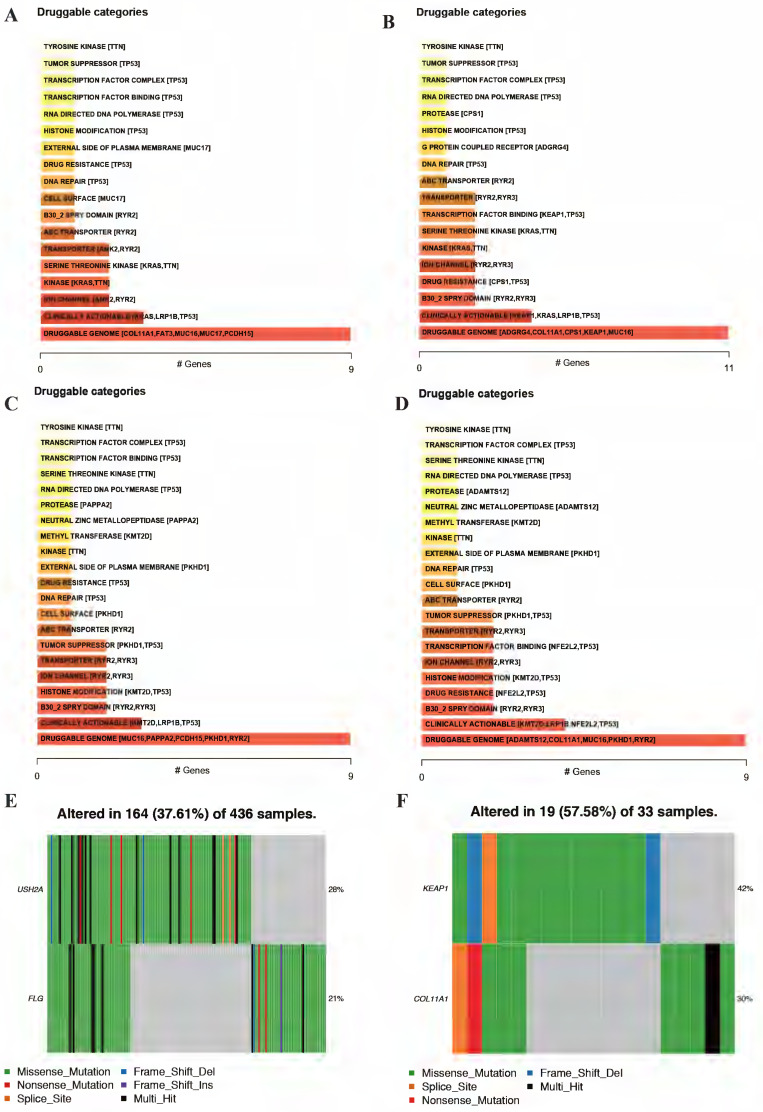
** Drug-gene interactions among the significant mutated genes in these 2 clusters.** Drug-gene interactions of top 5 mutated genes both in cluster 1 (A, C) and cluster 2 (B, D) of LUAD and LUSC, respectively. The plot shows potential druggable gene categories along with up to top 5 genes (if any) involved in them. Visualization of differentially mutated genes in cluster 1 (E) and cluster 2 (F) among NSCLC patients. LUAD, lung adenocarcinoma; LUSC, lung squamous cell carcinoma; NSCLC, non-small cell lung cancer.

After screening mutated oncogenic pathways, we also performed drug-gene interactions among the mutated top 5 genes in these 2 clusters (Figure 11A-D). As the results displayed, the plot shows potential druggable gene categories along with up to top 5 genes (if any) involved in them. Besides, the overview of differentially mutated genes according to cluster classification were visualized (Figure 11E-F). Meanwhile, the mutated gene interaction to drugs according to cluster classification were further explored (Table 3).

## Discussion

Autophagy underlying the initiation, progression, and metastasis of various cancers, including NSCLC. While aberrantly regulated autophagy in the prognosis of NSCLC and the mechanisms are less well defined. It reported that deregulation of UBE2C-mediated autophagy repression aggravates NSCLC progression 24. TRIM59 could as a new molecular biomarker for predicting the prognosis of NSCLC patients 24. The prognostic effect of circulating exosomes miR-425-3p on the response of NSCLC to platinum-based chemotherapy 26. In this study, we first screened 39 differentially expressed autophagy-related genes, then according to their prognostic value, 23 of 39 were related to the overall survival for NSCLC. Differ from regular analysis of cox and logistic regression, we classified the NSCLC into 2 subtypes according to consensus clustering based on the 23 prognostic genes. Besides, Kaplan-Meier analysis showed different prognosis between cluster 1 and cluster 2 subtypes. So according to these 2 clusters, DEGs of each cluster were identified to explore their biological functions.

According to our present study, we identified two autophagy-related gene subgroups using consensus clustering analysis based on 23 prognostic autophagy-related genes, in which we found cluster 1 have poor prognostic value in NSCLC. Considering the occurrence of mutation in NSCLC are very common, and the mutation site could be used as therapy target to improve the prognosis of NSCLC. At the same time, there are many studies focused on the relationship between mutation and immunotherapy, such as the epidermal growth factor receptor (EGFR) mutations, which are the second most common oncogenic driver event in NSCLC 27. Besides, Anti-PD1/PD-L1 immunotherapy has emerged as a standard of care for stage III-IV NSCLC over the past decade. Patient selection is usually based on PD-L1 expression by tumor cells and/or tumor mutational burden. While mutations in oncogenic driver genes will modify the immune tumor microenvironment and may promote anti-PD1/PD-L1 resistance 28. It is important to explore new mutation genes and investigated the biological functions to improve the prognosis. KMT2D mutation is associated with poor prognosis of NSCLC 29. Patients with LUSC gain overall favorable survival advantage from TTN mutation type, and overall favorable survival and disease-free survival advantage from TTN/TP53 double mutation 30. Patients with TP53/EGFR double mutations, especially missense mutations, have shorter response rates and PFS when treated with EGFR TKI 31. KRAS G12D and STK11 mutations confer poor prognoses for patients with KRAS-mutant NSCLC 32. In the current study, mutation profile signature showed that mutated FLG (21%), USH2A (28%) and KMT2D (22%) are special for cluster 1, mutated KEAP1 (42%), COL11A1 (30%) and XIRP2 (25%) are special for cluster 2.

Autophagy was involved in tumor microenvironment and the autophagy-related genes could affect the immune responses. There is evidence that the microenvironment of NSCLC is rich in different types of immune cells which are associated with clinical outcomes 33,34. The composition of the immune microenvironment differs across patients as well as in cancers of the same type. Early clinical studies revealed that immune cell infiltration had a major impact on the clinical course of several cancers 35-38. Thus, we also pooled immune cell infiltration signatures for each cluster. In cluster 1, T cells CD8, T cells CD4 memory resting, T cells CD4 memory activated, Macrophages M1, Dendritic cells resting, Mast cells resting, Monocytes, Dendritic cells activated, T cells follicular helper, B cells memory, T cells regulatory (Tregs) and Plasma cells have significant different infiltration levels. While Monocytes (p=0.035) and Macrophages M0 seems associated with cluster 2. Combined with previous studies, CD8 T cells are an important immune cell in tumor immune microenvironment. In addition to CD8, the importance of other subtypes of immune cells, including CD4 T cell and macrophage has also been reported. It has been reported that increased CD4 and CD8 T cell abundance in tumor immune microenvironment is associated with better survival outcomes 39,40. Macrophages are the most abundant cells, which performed several functions within the tumor microenvironment. Tumor-associated macrophages commonly refer to an alternative M2 phenotype, exhibiting anti-inflammatory and pro-tumoral effects. On the contrary, Macrophages M1 have pro-inflammatory effect and anti-tumoral effects 41. In the current study, we identified immune cell infiltration signatures based on TMB. The results showed that higher fractions of T cells CD4 memory resting, T cells follicular helper and Plasma cells in cluster 1 both in LUAD and LUSC, which maybe the reason why cluster 1 have worse prognosis. According to the results, it seems that the different immune cell infiltration signatures between cluster 1 and cluster 2 could influence the prognosis of NSCLC.

The oncogenic pathways involved in multiple cancers and related to the progression and prognosis of cancers, such as RTK-RAS, WNT, NOTCH, Hippo, PI3K, Cell Cycle, MYC, TGF-Beta and TP53 pathways. For instance, overexpression of Wnt-1, -2, -3, and -5a and of Wnt-pathway components Frizzled-8, Dishevelled, Porcupine, and TCF-4 is common in resected NSCLC and is associated with poor prognosis 42. Harmful NOTCH mutations are identified as new predictors of effective immunotherapy for NSCLC 43. YAP1 is the main Hippo pathway effector and an effective oncogene. It is overexpressed in NSCLC and the loss of YAP1 could be used as a clinical indicator to predict neuroendocrine characteristics and chemosensitivity 44. G3BP1 may play a key role in activating the PI3K/AKT/mTOR pathway and can be used as a new prognostic biomarker for patients with NSCLC undergoing surgery 45. KDM4A promotes the growth of NSCLC through Wnt/β-catenin signaling pathway and DLX5-mediated Myc expression 46. In our study, we found that the mutated oncogenic pathways are mainly involved in WNT, RTK-RAS, PI3K, NOTCH and Hippo signaling pathways in each cluster. The main difference between cluster 1 and cluster 2 is the mutated sample numbers, mutated gene numbers and types. It seems that cluster 1 have more mutated genes and sample numbers, caused the different activity of oncogenic pathways compared to cluster 2, which may contribute to the different prognosis of each cluster. Gene-drug interactions mainly divided into 3 categories, which including inhibitory interactions, induction interactions and phenoconversion interactions. Among them, inhibitory and induction interactions could affect the pharmacokinetics of drugs. Besides, these interactions can occur with the administration of a perpetrator drug that alters the drug metabolism or transport, as well as with the presence of loss- or gain-of-function genetic variants that alter function of enzymes 47. In the current study, we identified the potential druggable gene categories along with up to top 5 genes (if any) involved in them and found that Keap1 in cluster 2, could inhibited pharmacokinetics of bradoxolone methyl and dimethyl fumarate both in LUAD and LUSC. However, this needs to be further verified in the future. We believe our findings could provide newly insight in both classification and personalized treatment strategy.

In conclusion, based on autophagy-related gene expression characteristics, we used consensus clustering to identify 2 subtypes of NSCLC. These 2 subtypes shown significant different mRNA expression signatures and different immune cell infiltration patterns and mutation signatures. These differences may affect the tumor progression and tumor prognosis. This study may be helpful to accurately classify NSCLC patients and provide newly insight in personalized treatment.

## Supplementary Material

Supplementary figure.

## Figures and Tables

**Table 1:** Clinical characteristics of lung adenocarcinoma (LUAD) and lung squamous cell carcinoma (LUSC) patients in TCGA database

	LUAD	Percentage	LUSC	Percentage
Characteristic	N=486	%	N=504	%
Age (years)				
<65	209	43.00	170	33.73
≥65	258	53.09	325	64.48
Unknown	19	3.91	9	1.79
Gender				
Female	264	54.32	131	25.99
Male	222	45.68	373	74.01
T stage				
T1	163	33.54	114	22.62
T2	260	53.50	295	58.53
T3+T4	60	12.35	95	18.85
Unknown	3	0.61	-	-
N stage				
N0	312	64.20	320	63.49
N1	90	18.52	133	26.39
N2+N3	72	14.81	45	8.93
Unknown	12	2.47	6	1.19
M stage				
M0	333	68.52	414	82.14
M1	24	4.94	7	1.39
MX	125	25.72	79	15.68
Unknown	4	0.82	4	0.79

**Table 2:** Differentially expressed 39 autophagy-related genes in NSCLC

Gene	log FC	p	FDR
DLC1	-2.674452952	9.20E-58	8.78E-56
NRG3	-2.641312106	5.46E-53	1.64E-51
NLRC4	-2.213490722	1.89E-57	1.20E-55
DAPK2	-2.045550174	1.48E-52	3.53E-51
MAP1LC3C	-1.900957933	1.54E-43	1.40E-42
CCL2	-1.894463992	2.32E-18	5.76E-18
HSPB8	-1.890412952	5.99E-53	1.64E-51
FOS	-1.8678626	9.20E-32	4.39E-31
PPP1R15A	-1.673892269	2.70E-45	3.43E-44
GRID1	-1.500483661	3.10E-45	3.70E-44
DRAM1	-1.441257298	7.12E-44	6.80E-43
PRKCQ	-1.398259164	1.40E-45	2.06E-44
DAPK1	-1.344133398	4.13E-43	3.59E-42
ITPR1	-1.000224806	1.32E-38	1.01E-37
ATG4D	1.001394367	2.46E-28	1.02E-27
BAK1	1.053922698	6.60E-40	5.25E-39
DDIT3	1.055361827	8.16E-20	2.29E-19
EIF4G1	1.07484107	5.82E-34	3.37E-33
IFNG	1.096213275	0.02578937	0.027214197
HDAC1	1.133747556	1.69E-52	3.59E-51
P4HB	1.161987407	2.06E-52	3.94E-51
FADD	1.204458771	9.69E-42	8.04E-41
EGFR	1.222356033	2.98E-07	4.42E-07
PARP1	1.236206553	1.31E-50	2.27E-49
ATIC	1.258107209	5.21E-56	2.49E-54
SPHK1	1.276199145	1.53E-15	3.39E-15
BNIP3	1.365963835	1.32E-33	7.41E-33
TP73	1.474554334	9.12E-14	1.85E-13
IKBKE	1.475598986	2.04E-45	2.79E-44
PTK6	1.764962137	8.88E-19	2.32E-18
ATG9B	1.979798346	2.42E-21	7.10E-21
TMEM74	2.161296281	4.04E-08	6.28E-08
GAPDH	2.346547707	3.42E-55	1.31E-53
ITGB4	2.49728055	1.54E-33	8.43E-33
EIF4EBP1	2.547118173	2.41E-44	2.42E-43
NKX2-3	3.395091393	6.47E-10	1.12E-09
CDKN2A	4.149641931	4.52E-20	1.31E-19
BIRC5	4.429533634	1.28E-58	2.44E-56
TP63	5.075495827	3.63E-09	6.08E-09

NSCLC, non-small cell lung cancer.

**Table 3:** Drug-gene interactions of significant mutated genes in each cluster.

Gene	Cluster	Interaction types	Drug name	Drug claim name
KEAP1	2	inhibitor	BARDOXOLONE METHYL	CHEMBL1762621
KEAP1	2	inhibitor	DIMETHYL FUMARATE	CHEMBL2107333
COL11A1	2	-	OCRIPLASMIN	CHEMBL2095222
COL11A1	2	-	COLLAGENASE CLOSTRIDIUM HISTOLYTICUM	CHEMBL2108709
FLG	1	-	PROPIONIBACTERIUM	ACNES
KMT2D	1	-	BICALUTAMIDE	Bicalutamide
