# Supplementary material for: Autophagy-related tumor subtypes associated with significant gene expression profiles and immune cell infiltration signatures to reveal the prognosis of non-small cell lung cancer
Source: J Cancer. 2023 May 15;14(8):1427–42. doi: 10.7150/jca.83097 (PMC10240669; doi:10.7150/jca.83097)
Supplement: Supplementary file 1 — Supplementary figure. [file jcav14p1427s1.pdf]

Supplementary Figure S1. Relationship between autophagy-related genes and prognosis of NSCLC. There are 23 autophagy-related genes (A-W) have prognostic value according to the Kaplan Meier-plotter database. NSCLC, non-small cell lung cancer.

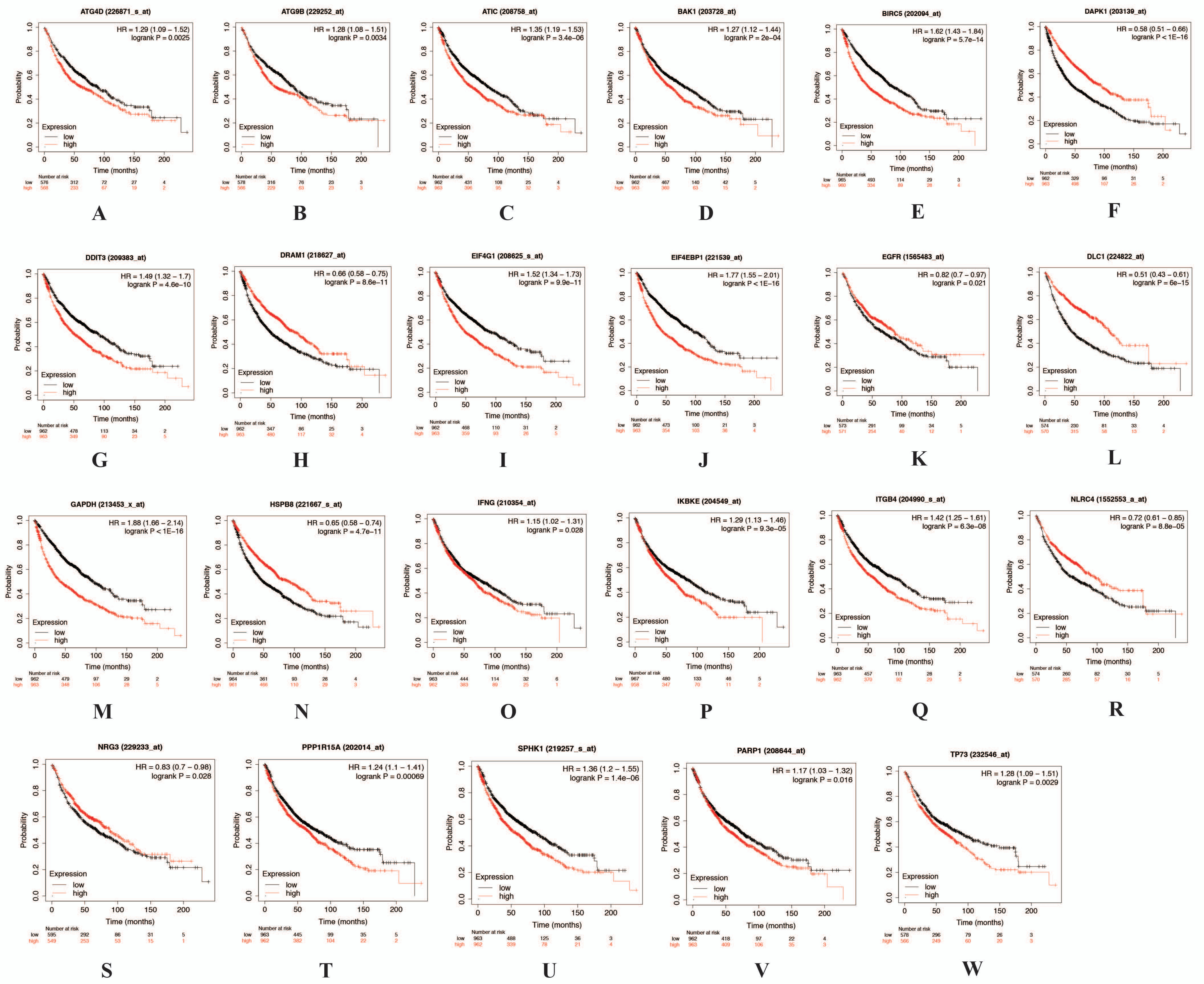
ATG4D (226871_s_at)
HR = 1.29 (1.09 – 1.52)
logrank P = 0.0025
A
ATG9B (229252_at)
HR = 1.28 (1.08 – 1.51)
logrank P = 0.0034
B
ATIC (208758_at)
HR = 1.35 (1.19 – 1.53)
logrank P = 3.4e−06
C
BAK1 (203728_at)
HR = 1.27 (1.12 – 1.44)
logrank P = 2e−04
D
BIRC5 (202094_at)
HR = 1.62 (1.43 – 1.84)
logrank P = 5.7e−14
E
DAPK1 (203139_at)
HR = 0.58 (0.51 – 0.66)
logrank P < 1E−16
F
DDIT3 (209383_at)
HR = 1.49 (1.32 – 1.7)
logrank P = 4.6e−10
G
DRAM1 (218627_at)
HR = 0.66 (0.58 – 0.75)
logrank P = 8.6e−11
H
EIF4G1 (208625_s_at)
HR = 1.52 (1.34 – 1.73)
logrank P = 9.9e−11
I
EIF4EBP1 (221539_at)
HR = 1.77 (1.55 – 2.01)
logrank P < 1E−16
J
EGFR (1565483_at)
HR = 0.82 (0.7 – 0.97)
logrank P = 0.021
K
DLC1 (224822_at)
HR = 0.51 (0.43 – 0.61)
logrank P = 6e−15
L
GAPDH (213453_x_at)
HR = 1.88 (1.66 – 2.14)
logrank P < 1E−16
M
HSPB8 (221667_s_at)
HR = 0.65 (0.58 – 0.74)
logrank P = 4.7e−11
N
IFNG (210354_at)
HR = 1.15 (1.02 – 1.31)
logrank P = 0.028
O
IKBKE (204549_at)
HR = 1.29 (1.13 – 1.46)
logrank P = 9.3e−05
P
ITGB4 (204990_s_at)
HR = 1.42 (1.25 – 1.61)
logrank P = 6.3e−08
Q
NLRC4 (1552553_a_at)
HR = 0.72 (0.61 – 0.85)
logrank P = 8.8e−05
R
NRG3 (229233_at)
HR = 0.83 (0.7 – 0.98)
logrank P = 0.028
S
PPP1R15A (202014_at)
HR = 1.24 (1.1 – 1.41)
logrank P = 0.00069
T
SPHK1 (219257_s_at)
HR = 1.36 (1.2 – 1.55)
logrank P = 1.4e−06
U
PARP1 (208644_at)
HR = 1.17 (1.03 – 1.32)
logrank P = 0.016
V
TP73 (232546_at)
HR = 1.28 (1.09 – 1.51)
logrank P = 0.0029
W
Probability
Time (months)
Expression
low
high
Number at risk
